# Development of a blood test for uterine sarcoma—Diagnosis and monitoring (DOORS-D and DOORS-M) studies

**DOI:** 10.1371/journal.pone.0339336

**Published:** 2026-02-17

**Authors:** Anna Casey, Rebecca Allsopp, Jacqui Shaw, Caroline Cowley, Elizabeth Stannard, Yvette Griffin, Indrajeet Das, Marc Wadsley, Natalie Darko, Esther L. Moss

**Affiliations:** 1 Leicester Cancer Research Centre, College of Life Sciences, University of Leicester, Leicester, United Kingdom; 2 University Hospitals of Leicester NHS Trust, Infirmary Square, Leicester, United Kingdom; 3 College of Life Sciences, University of Leicester, Leicester, United Kingdom; The University of Texas MD Anderson Cancer Center, UNITED STATES OF AMERICA

## Abstract

Uterine sarcomas can be difficult to differentiate from uterine fibroids due to many shared symptoms and imaging features, which can result in delayed or missed diagnosis, or over treatment. The ‘Development of a blood test for uterine Sarcoma – Diagnosis’ (DOORS-D) (ISRCTN14800787) and ‘Development of a blood test for uterine Sarcoma – Monitoring’ (DOORS-M) (ISRCTN14174468) studies aim to explore the role for circulating tumour DNA (ctDNA) to diagnose and to monitor uterine sarcomas. DOORS-D will recruit patients who have a suspected uterine sarcoma or large fibroid and are due to undergo surgery (hysterectomy or myomectomy) for a blood sample prior to surgery whereas DOORS-M will recruit patients who have been diagnosed with a uterine sarcoma in the previous 10 years for longitudinal blood sampling every 3–6 months over the course of the study. Information will be collated on patient characteristics and symptoms, tumour characteristics and diagnostic imaging, with representative images selected and analysed using large language models. Analysis of genomic/methylation profile of ctDNA samples collected from DOORS-M will be used to design a ctDNA-based ‘test’. Analysis of the samples collected from the participants recruited to the DOORS-D study will enable the accuracy of the ‘test’ to differentiate uterine sarcomas from fibroids to be determined. In addition, the opinions of patients with a suspected or confirmed sarcoma will be explored through semi-structured qualitative interviews. Purposeful recruitment strategy will ensure that the experiences of women from diverse socioeconomic, cultural and ethnic backgrounds are included. The results of the studies will be shared through conference presentations and peer-reviewed publications.

## Introduction

Uterine sarcomas are rare mesenchymal tumours that account for 3% of all uterine cancers and are often associated with a poor long-term prognosis [1]. Uterine sarcomas typically arise from myometrial smooth muscle or endometrial stroma, with leiomyosarcomas (LMS) being the most common subtype accounting for 60–70% of cases, followed by endometrial stromal sarcomas (ESS) at 10–20% [2]. Their myometrial, rather than endometrial, location makes diagnosis more challenging since biopsy of the endometrium is only able to detect 35% of LMS and 25% of ESS [3]. The other key diagnostic challenge is the difficulty differentiating a uterine sarcoma from uterine fibroids which are very common benign uterine growths affecting 40–80% of women [4], due to shared symptomatology [5]. Fibroids especially impact women aged 40–60 years, who have a 4–11 times greater risk than younger women (20–30 years) or older women (>60 years) [6]. Women of African ancestry also experience a higher incidence and prevalence of uterine fibroids compared to women from other races or ethnic groups, 30.6/1000 women-years compared to 8.9/1000 for White women [7].

Accurately differentiating between a uterine fibroid and a sarcoma is of paramount importance, not only to expediate oncology management of patients with a sarcoma, but to avoid procedures that could cause result in cancer dissemination and thereby worsening patients’ prognosis. Although the incidence of unexpected sarcoma at uterine fibroid surgery is low, 0.175 to 0.67% [5], it has been reported that 19% of unexpected sarcoma cases underwent a subtotal hysterectomy or open myomectomy [8]. Intraoperative pathology assessment can help with guiding patient management however, this is can be associated with diagnostic challenges [9], and appears to be less concordant with final histology compared to assessment of other gynaecological malignancies [10]. Laparoscopic myomectomy with morcellation in particular, is associated with a significantly lower 5-year recurrence-free survival rate compared to total hysterectomy for unexpected uterine sarcoma, 24.1% compared to 43.6%, with greater rates of peritoneal dissemination reported, 22.2% versus 0.0% [11]. Consequentially, the United States Food and Drug Administration (FDA) have advised morcellation within retrieval bags to prevent dissemination [12,13].

At present there is no specific blood test that can be used pre-operatively to differentiate between uterine sarcomas or fibroids. The tumour marker CA125 is not helpful [14]. LDH and its isoform LDH3 have been shown to be an independent predictor of LMS [15] although not validated for clinical use, and obesity can be a confounding factor increasing the false positive rate [16]. Other blood markers, such as C-reactive protein and absolute neutrophil count, have shown associations with sarcomas [17] however lack specificity. Imaging, in particular MRI, can be used to differentiate between uterine sarcomas and fibroids with a high level of diagnostic accuracy [18]. Features including peritoneal or lymph node metastases, high/intermediate on T2-weighted imaging, low apparent diffusion coefficient (ADC) and high intensity signal of diffusion weighted images were identified as having a strong association with uterine sarcomas [19]. Other features, such an irregular margin, haemorrhage and enhancement, were determined as being an indeterminate association [19], and it is reported that there is a greater challenge when differentiating degenerating or complex fibroids from uterine sarcomas [20]. PET-CT scans have been shown to have a greater ability to distinguish fibroids from sarcomas, sensitivity of 80.8% and specificity of 100% [21], however, such imaging would typically only be performed in cases where there was a high suspicion of sarcoma, therefore is unlikely to impact the unexpected sarcoma diagnosis rate.

The genomic profile of uterine sarcomas reveals a mixed picture with common somatic mutations, including *TP53* (56%) and *RB1* (51%) but also somatic copy number alterations (SCNAs), with whole genome duplication (WGD) identified in half of high-grade sarcomas [22]. Uterine fibroids are monoclonal tumours although 40–50% are reported to have detectable chromosomal abnormalities, and a recent genome wide meta-analysis has identified 46 novel genes associated with fibroids [23]. Genomic analysis, for example, shallow whole genome sequencing (sWGS) shows potential to discriminate between sarcomas and fibroids, with a pilot study reporting that the percentage of the genome affected by SCNAs in plasma of six patients with fibroids was lower than the proportion of the genome showing SCNA in patients with sarcoma [24].

Liquid biopsies based on circulating tumour DNA (ctDNA) are rapidly becoming established as a minimally invasive tool for diagnosis, guiding treatment regimens and monitoring for recurrence [25]. The evidence to support a role in the monitoring and detection of endometrial cancer recurrence is increasing [26–29] with a higher diagnostic accuracy compared to the currently available tumour markers, CA125 and HE4 [30]. The potential of the ‘liquid biopsy’ to monitor uterine sarcomas has been explored in previous studies where ctDNA has shown promise as a marker both in the monitoring [31–33] and diagnosis [24] of uterine sarcomas, although other analytes including microRNAs have also been shown to have potential as biomarkers [34]. Leiomyosarcomas were the most common histological subtype investigated and there was significant heterogeneity in terms of both laboratory approaches and the reporting of these approaches. Given the small number of uterine sarcoma cases in the majority of the studies to date, the proportion of cases with detectable ctDNA is currently unknown, and there is no cost-effective test that could give a result in a short time frame to guide patient management, thereby reducing pre-operative diagnostic uncertainty.

### Hypothesis

ctDNA is an acceptable test that can be used in routine clinical practice to diagnose and/or monitor uterine sarcomas.

## Materials and methods

### Ethical approval and trial registration

In order to address the two potential roles for ctDNA, two studies have been designed: Development of a blood test for uterine Sarcoma – Diagnosis (DOORS-D) and Development of a blood test for uterine Sarcoma – Monitoring (DOORS-M). Both studies have received ethical approval from the South Central – Berkshire B Research Ethics Committee (25/SC/0107 and 25/SC/0108) and have been registered on ISRCTN registry (https://www.isrctn.com/ISRCTN14800787 registered on 03/06/2025 and https://www.isrctn.com/ISRCTN14174468 registered on 04/06/2025). The study is being conducted by academics and clinicians with expertise in gynaecological oncology (EM), liquid biopsies and genomic analysis (JS/RA/CC/MW), pathology (ES), radiology (YG/ID) and health equity (ND). The study is sponsored by the University of Leicester (protocol numbers 1040 (DOORS-M), 1041 (DOORS-D) and opened for recruitment in July 2025. The clinical research teams will be notified of protocol modifications through the sponsor’s research governance team. The research team (EM/JS/CC/MW/ND) are employees of the study sponsor and have designed of the study and will conduct, analyse, and report the outcomes. Given the nature of the studies a Data Monitoring Committee will not be required, instead representatives from the sponsor’s research governance team will monitor the conduct of the study and ensure that all procedures and requirements are adhered to. Participant recruitment will continue until 31/01/2028 and data collection will be completed by 28/03/2028 (end of study), with additional follow-up data on patient outcomes collected up to 2 years following the end of the study. It is anticipated that the results of the qualitative aspects of the studies will be reported in 2026, whereas results from the clinical aspect of the studies will be reported in 2028.

### Public and patient involvement (PPI)

PPI groups and outreach events have been held with women from diverse ethnic backgrounds, including of Black ethnicity, to discuss the challenge of uterine fibroid management and the need to identify uterine sarcomas in a timely manner. The PPI group reported awareness of uterine fibroids but not sarcomas, and were supportive of the development of a blood test to guide diagnosis and management. The study design is similar to a previous cohort study developing a blood test to diagnose endometrial cancer recurrence, which was deemed a very acceptable test by the study participants [35]. A PPI group to advise the research team during the conduct of the study will be recruited.

### Study design

DOORS-D is a single centre case series study that is being conducted in an academic gynaecological oncology centre at the University Hospitals of Leicester. DOORS-D aims to investigate whether genomic alterations in plasma are able to distinguish between uterine sarcomas and uterine fibroids, with a secondary aim to investigate the potential of radiomics and large language models to diagnose sarcomas.

DOORS-M is a cohort study, collecting data and longitudinal samples from patients who have been diagnosed with a uterine sarcoma that aims to create genomic and methylation profiles for uterine sarcomas to identify signatures that could be used within a ctDNA ‘test’ to detect or predict recurrence. The lead recruitment site will be the University Hospitals of Leicester, Additional recruitment sites will be established however, no blood samples will be collected from the other sites, instead information on patient characteristics, archived tumour samples and sequencing results, and imaging will be collected.

Both studies include secondary objectives of exploring patients’ opinions and the psychological impact of ctDNA to diagnose/monitor uterine sarcomas. Table 1 includes the study objectives for the two studies.

**Table 1:** Objectives for DOORS-D and DOORS-M studies.

DOORS-D	
Primary objective	To investigate whether genomic alterations in plasma are able to distinguish between uterine sarcomas and fibroids
Secondary objectives	To investigate the ability of a ctDNA ‘test’ to detect uterine sarcomas To investigate the potential of Artificial Intelligence (AI) to diagnose uterine sarcomas To explore the psychological impact of ctDNA testing to guide uterine sarcoma diagnosis
**DOORS-M**	
Primary objective:	To create genomic and methylation profiles for uterine sarcomas
Secondary objectives:	To investigate the ability of a ctDNA ‘test’ to detect uterine sarcoma recurrence To investigate the potential of AI to diagnose uterine sarcomas To explore the psychological impact of ctDNA testing in uterine sarcomas follow-up To identify a symptom profile associated with uterine sarcomas

### Sample size

DOORS-D and DOORS-M both aim to recruit 50 patients. Sample size calculations for DOORS-D have determined that for a cohort of 50 patients a 40% prevalence of uterine sarcoma (20 cases) would give a 95% confidence interval of width 0.247 for a sensitivity of 0.95 (95% CI: 0.751–0.999). DOORS-M aims to recruit 50 patients with a previous diagnosis of uterine sarcoma. Given the high rate of uterine sarcoma recurrence, recruitment will be achievable within the study duration, given that the University Hospitals of Leicester currently discusses more than 50 patients with ‘suspicious looking fibroid, sarcoma cannot be excluded’ in the multidisciplinary team meetings per year of which 7–10 are confirmed to be a sarcoma on histology examination.

### Study population

DOORS-D will recruit women due to undergo surgery, either hysterectomy or myomectomy, for a suspected uterine sarcoma or a fibroid, whereas DOORS-M will recruit patients who have already received a uterine sarcoma diagnosis within the previous 10 years. Adult females aged 18–99 years, who meet the above criteria, are willing and able to consent to participate in the trial, and understand study requirements, with interpreter support if needed, will be offered recruitment.

### Consent procedures

Potential participants will be given a participant information sheet, the opportunity to ask questions, and be given at least 24 hours decision time before consent is taken. Written consent will be taken by a member of the clinical research team and will include a discussion on the aims and objectives of the study, the study processes and potential risks. It will be explained to the potential participant that they are free to withdraw at any time, they are not obliged to give a reason, and that this will not impact on their future care. Women who do not have English as their primary language will be offered an interpreter to support the consent process.

### Study processes – DOORS-D

**Fig 1:**
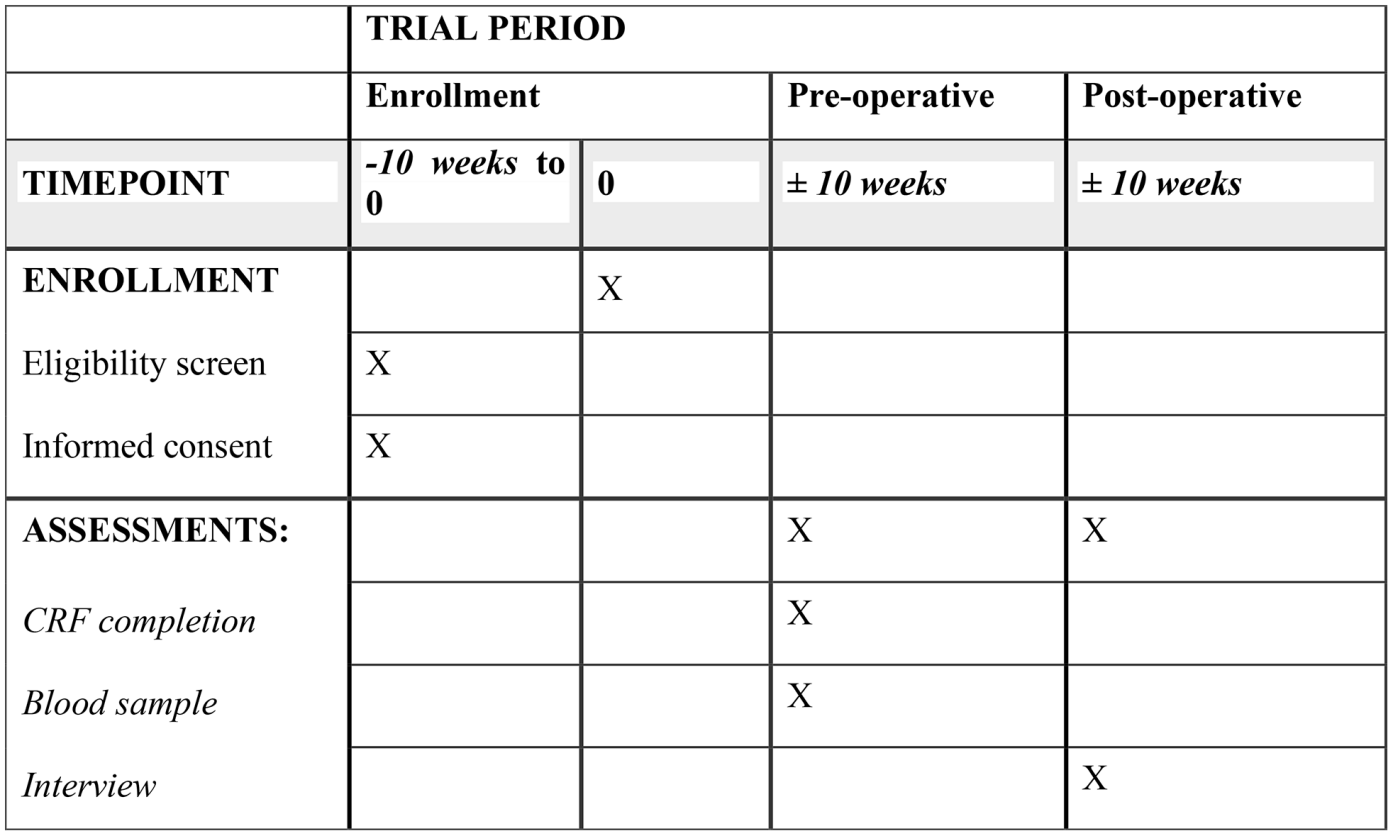
Participant timeline: Schedule of enrolment, interventions, and assessments for DOORS-D.

The schedule of enrolment, interventions and assessment for DOORS-D are described in Fig 1 [36].

Participants recruited to DOORS-D study will have a Case Report Form (CRF) completed detailing their demographic characteristics, medical history, and current clinical episode. Pre-operative radiological imaging, including ultrasound scans, CT/MR/PET scans will undergo a blinded review by the study radiologist, and representative images selected for future AI analysis. The analysis plan using radiomics and large language models has not been determined *a priori* due to rapid changes within the field. Prior to surgery 20mls of blood will be collected, processed into plasma and stored for batched ctDNA analysis. The histological diagnosis from surgery will be classified into 1) uterine fibroid; 2) uterine sarcoma; or 3) smooth muscle tumour of uncertain malignant potential (STUMP). A blinded pathological review will be undertaken by the study pathologist to confirm the diagnosis. Participants diagnosed with a sarcoma or STUMP will be offered recruitment to the DOORS-M study. DNA will be extracted from pathology blocks, either fresh or formalin fixed, for whole exome sequencing (WES). Following surgery, a subset of participants will be invited to an interview to discuss their experiences of the study. Recruitment will be purposeful in order to ensure a breadth of opinions from participants from diverse age, socioeconomic and ethnic populations. The interviews will be transcribed verbatim using Microsoft Teams and will be analysed using a reflexive thematic analysis, as described by Braun and Clarke [37].

### Study processes – DOORS-M

**Fig 2:**
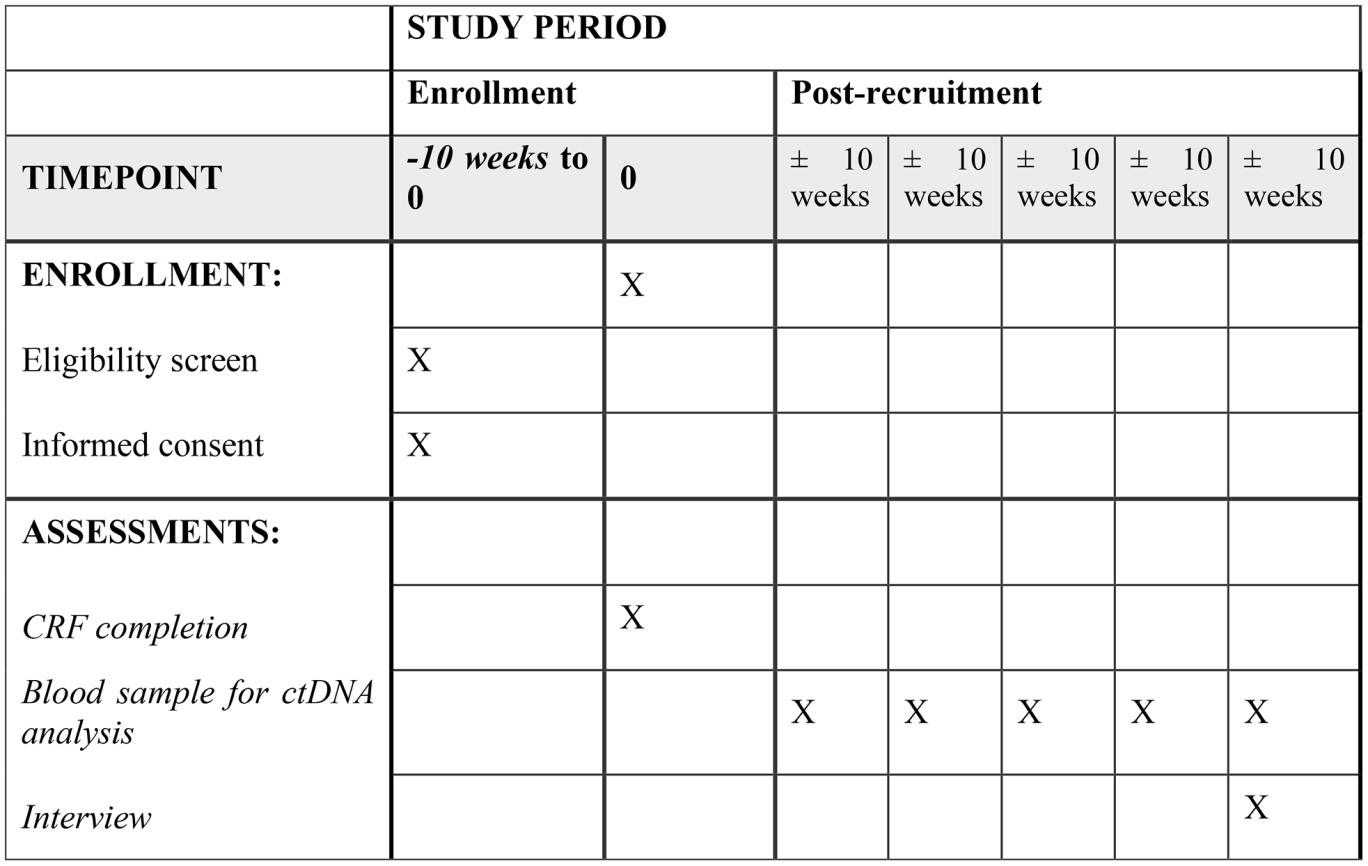
Participant timeline: Schedule of enrollment, interventions, and assessments for DOORS-M study.

The schedule of enrolment, interventions and assessment for DOORS-M are described in Fig 2.

Potential participants will be identified through their clinical care team, screened, and if eligible approached by a member of the research team for recruitment. Once recruited, information will be collected on a CRF to collate information on the patient’s demographics, previous medical history and sarcoma diagnosis/treatment. Tumour blocks from previous diagnostic or treatment biopsies will be requested for DNA extraction and WES, unless previously sequenced as part of the patient’s clinical care. A blinded pathological review will be undertaken by the study pathologist to confirm the diagnosis. Pre-operative imaging will undergo a blinded review following the same process outlined in DOORS-D, and representative images stored for future AI analysis. At recruitment the participant will have 20mls of blood collected for processing into plasma and storage for ctDNA analysis. Given the constant advances in the field we have not predetermined *a priori* the ctDNA analysis technique. Further blood samples will be collected at subsequent clinic appointments, or 3–6 monthly, for as long as the patient remains within the study. Only participants recruited at the University Hospitals of Leicester will undergo blood sampling due to logistic and financial constraints however, participants will consent to analysis of archival tumour tissue for DNA extraction, thereby expanding the number of sarcoma cases available for analysis. Recruitment from other sites will also involve CRF completion and participant interviews, thereby extending the geographical spread of the study. As with DOORS-D, a subset of participants will be recruited for a semi-structured qualitative interview to discuss their opinions and the potential psychological implications of using ctDNA to diagnose, and/or predict, sarcoma recurrence. The selection of participants will aim to capture a range of opinions and experiences and include an ethnically and socioeconomically diverse population.

### Safety considerations

The DOORS-D and DOORS-M studies are data/sample collection studies and the only physical study procedure participants will be undertaking is venepuncture. The qualitative interview may bring up upsetting memories and participants will be advised that they may choose not to answer a question if they do not wish to. The research team undertaking the interviews are experienced in conducting interviews with oncology populations, following a distress protocol when indicated, supporting participants, and signposting to additional support in order to minimise any distress, including clinical nurse specialists and cancer charity support. Should analysis of a participant’s tumour, radiology, and/or ctDNA result in the identification of an abnormality or genetic mutation that could impact the patient, or their family members, for example BRCA mutations or Lynch syndrome, this result will be communicated to the patient’s clinical care team for onward investigation and if advised further investigations or referrals.

### Analysis

Data from the two studies will be collected from all the participants using the CRFs and descriptive statistics used to report patient demographics and tumour subtype characteristics. Missing data will be minimised by making efforts to ensure complete follow-up. Given the study design all available data will be included and participants will be censored at their last clinic appointment. Correlation between the second pathology and radiology reviews and the patient’s original diagnosis will be undertaken (kappa coefficient). Genomic and/or methylation profiles generated from the study data will be descriptive. Descriptive statistics will be used to report the sensitivity, specificity and diagnostic accuracy of ctDNA and large language model analysis of radiological images to differentiate uterine sarcomas from uterine fibroids. The qualitive interviews will be analysed using a reflexive thematic analysis, as described by Braun and Clarke [37].

### Study timelines

The studies opened at the University Hospitals of Leicester on 14/07/2025 and will continue recruiting until 29 February 2028. Results from the qualitative study will be expected after 1 year of recruitment, whereas the results from the AI analysis of radiological imaging and genomic analysis will be expected following completion of recruitment.

## Discussion

The DOORS-D and DOORS-M studies aim to explore the potential of a ctDNA-based approach to uterine sarcoma management and have the potential to give greater insights into the landscape and challenges of differentiating uterine sarcomas from uterine fibroids. Each study aims to recruit 50 patients, to attain 20 sarcoma cases (40% prevalence) in DOORS-D and 50 cases in DOORS-M. Although this is a large number of cases for a rare malignancy, there is the possibility that our cohort may not include rarer subtypes. The developed ctDNA test through these studies would be trialled in a larger uterine sarcoma population, thereby determining diagnostic ability and enabling test modification. Such an approach has been explored previously [24] however, as yet there is no test that can be used in everyday clinical practice to guide patient management. Also, there is a need for greater understanding of the genomic landscape of sarcomas from women of Black ethnicity to ensure that any test that is developed has equitable diagnostic accuracy, unlike other tests currently used to diagnose endometrial cancer, for example the transvaginal ultrasound scan which has a lower diagnostic ability in women of Black ethnicity [38]. The studies include qualitative interviews to ensure that patients’ voices and opinions are heard and are able to contribute to the future development of blood test diagnosis or monitoring for uterine sarcomas. In addition, exploration of opinions and experiences of women from ethnic minority populations is essential in determining the potential impact for such a ‘test’ due to the significantly poorer long-term uterine cancer survival rates [39] and greater prevalence of uterine fibroids [7]. Previous work that the research team has undertaken, identified a lack of awareness of uterine malignancy amongst women from Black ethnic populations resident in England [40] and although efforts are being undertaken to raise awareness of the signs and symptoms of uterine cancer [41, 42], these focus typically on the symptom profile associated with endometrial cancer rather than uterine sarcomas.

There is a clear need to avoid undertreatment and potentially disseminating surgical procedures, however, this needs to be balanced with patients’ wishes for fertility preservation, especially in younger women [11]. One of the main challenges is that over half of patients diagnosed with an unexpected uterine sarcoma are pre-menopausal and/or under the age of 50 years, and the common presenting symptoms, irregular uterine bleeding, pelvic mass and menorrhagia, are indistinguishable from symptoms associated with uterine fibroids [5]. A percutaneous core needle biopsy is routinely performed to diagnose suspected soft-tissue sarcomas [43] however, this is an invasive procedure and although the reported dissemination rate is low for retroperitoneal sarcomas [44] there is no data for uterine sarcomas. It also would require a suspicion of sarcoma and therefore may not impact unexpected cases. It also requires clinicians with the relevant skills to perform the procedure, which is unlikely to be available at every hospital, thereby requiring the patient to travel to a centre with the necessary expertise. The potential of a ctDNA test that would negate the need for a biopsy and could reduce the duration of the patient’s diagnostic pathway. In addition, genomic information on an individual’s sarcoma could potentially be used to guide their management, particularly for immunotherapy, since a number of uterine sarcomas have been identified as having microsatellite instability (MSI) or deletions of *PDCD1*, which encodes PD-1, as well as other actionable mutations such as in *BRCA2* (5%), *BRAF* and *ALK* fusions [22, 45].

Although features associated with uterine sarcomas have been described radiologically [46], the accuracy of differentiating sarcomas from degenerating uterine fibroids using morphological features on MRI is variable, even by expert radiologists [47], sensitivity 70.7% and specificity 76.2% [20]. Radiomic analysis of radiological images may improve diagnostic accuracy with imaging [48, 49] and help reduce intra-observer variability.

In conclusion, the DOORS-D and DOORS-M studies aim to add to the genomic data on uterine sarcomas from a diverse ethnic population, the challenge of correctly differentiating uterine sarcomas from uterine fibroids, and exploring the experiences and opinions of patients who have undergone surgery for a suspected or are confirmed to have a uterine sarcoma.

## Supporting information

S1 Checklist SPIRIT checklist. (DOCX)

## References

[1] Amant F, Coosemans A, Debiec-Rychter M, Timmerman D, Vergote I. Clinical management of uterine sarcomas. Lancet Oncol. 2009;10(12):1188–98. doi: 10.1016/S1470-2045(09)70226-8 19959075

[2] Bogani G, Caruso G, Ray-Coquard I, Ramirez PT, Concin N, Ngoi NY, et al. Uterine leiomyosarcoma. Int J Gynecol Cancer. 2025;35(9):101992. doi: 10.1016/j.ijgc.2025.101992 40743869

[3] Hinchcliff EM, Esselen KM, Watkins JC, Oduyebo T, Rauh-Hain JA, Del Carmen MG, et al. The Role of Endometrial Biopsy in the Preoperative Detection of Uterine Leiomyosarcoma. J Minim Invasive Gynecol. 2016;23(4):567–72. doi: 10.1016/j.jmig.2016.01.022 26851414

[4] Pavone D, Clemenza S, Sorbi F, Fambrini M, Petraglia F. Epidemiology and Risk Factors of Uterine Fibroids. Best Pract Res Clin Obstet Gynaecol. 2018;46:3–11. doi: 10.1016/j.bpobgyn.2017.09.004 29054502

[5] Yorgancı A, Meydanlı MM, Kadıoğlu N, Taşkın S, Kayıkçıoğlu F, Altın D, et al. Incidence and outcome of occult uterine sarcoma: A multi-centre study of 18604 operations performed for presumed uterine leiomyoma. J Gynecol Obstet Hum Reprod. 2020;49(1):101631. doi: 10.1016/j.jogoh.2019.101631 31499285

[6] Baird DD, Dunson DB. Why is parity protective for uterine fibroids?. Epidemiology. 2003;14(2):247–50. doi: 10.1097/01.EDE.0000054360.61254.27 12606893

[7] Marshall LM, Spiegelman D, Barbieri RL, Goldman MB, Manson JE, Colditz GA, et al. Variation in the incidence of uterine leiomyoma among premenopausal women by age and race. Obstet Gynecol. 1997;90(6):967–73. doi: 10.1016/s0029-7844(97)00534-6 9397113

[8] Wais M, Tepperman E, Bernardini M, Gien L, Jimenez W, Murji A. A Multi-Center Retrospective Review of Clinical Characteristics of Uterine Sarcoma. J Minim Invasive Gynecol. 2015;22(6S):S93. doi: 10.1016/j.jmig.2015.08.250 27679381

[9] Lok J, Tse KY, Lee EYP, Wong RWC, Cheng ISY, Chan ANH, et al. Intraoperative Frozen Section Biopsy of Uterine Smooth Muscle Tumors: A Clinicopathologic Analysis of 112 Cases With Emphasis on Potential Diagnostic Pitfalls. Am J Surg Pathol. 2021;45(9):1179–89. doi: 10.1097/PAS.0000000000001746 34074809

[10] Hagen JA, Tessier K, Khalifa M, Rivard CL. Accuracy of Frozen Section Analysis in the Intraoperative Diagnosis of Uterine Leiomyosarcoma: Experience at a Large Metropolitan Hospital. Journal of the American College of Surgeons. 2021;233(5):S162. doi: 10.1016/j.jamcollsurg.2021.07.321

[11] Gao Z, Li L, Meng Y. A Retrospective Analysis of the Impact of Myomectomy on Survival in Uterine Sarcoma. PLoS One. 2016;11(2):e0148050. doi: 10.1371/journal.pone.0148050 26828206 PMC4735478

[12] Einarsson JI, Cohen SL, Fuchs N, Wang KC. In-bag morcellation. J Minim Invasive Gynecol. 2014;21(5):951–3. doi: 10.1016/j.jmig.2014.04.010 24769447

[13] US Food and Drug Administration 2023. Accessed 09.02.2026 https://www.fda.gov/medical-devices/surgery-devices/laparoscopic-power-morcellators

[14] Yilmaz N, Sahin I, Kilic S, Ozgu E, Gungor T, Bilge U. Assessment of the predictivity of preoperative serum CA 125 in the differential diagnosis of uterine leiomyoma and uterine sarcoma in the Turkish female population. Eur J Gynaecol Oncol. 2009;30(4):412–4. 19761133

[15] Goto A, Takeuchi S, Sugimura K, Maruo T. Usefulness of Gd-DTPA contrast-enhanced dynamic MRI and serum determination of LDH and its isozymes in the differential diagnosis of leiomyosarcoma from degenerated leiomyoma of the uterus. Int J Gynecol Cancer. 2002;12(4):354–61. doi: 10.1046/j.1525-1438.2002.01086.x 12144683

[16] Spivack LE, Glantz JC, Lennon C, Bhagavath B. Specificity of the lactate dehydrogenase isoenzyme index as a preoperative screen for uterine sarcoma before myomectomy. Fertil Steril. 2021;115(1):174–9. doi: 10.1016/j.fertnstert.2020.07.020 33070962

[17] Suh DS, Song YJ, Roh H-J, Lee SH, Jeong DH, Lee TH, et al. Preoperative Blood Inflammatory Markers for the Differentiation of Uterine Leiomyosarcoma from Leiomyoma. Cancer Manag Res. 2021;13:5001–11. doi: 10.2147/CMAR.S314219 34211296 PMC8239166

[18] Raffone A, Raimondo D, Neola D, Travaglino A, Giorgi M, Lazzeri L, et al. Diagnostic accuracy of MRI in the differential diagnosis between uterine leiomyomas and sarcomas: A systematic review and meta-analysis. Int J Gynaecol Obstet. 2024;165(1):22–33. doi: 10.1002/ijgo.15136 37732472

[19] Hindman N, Kang S, Fournier L, Lakhman Y, Nougaret S, Reinhold C, et al. MRI Evaluation of Uterine Masses for Risk of Leiomyosarcoma: A Consensus Statement. Radiology. 2023;306(2):e211658. doi: 10.1148/radiol.211658 36194109 PMC9885356

[20] Loughborough WW, Rockall AG, Gagliardi TT, Satchwell L, Greenlay E, Osborne P, et al. Comparison of MRI imaging features to differentiate degenerating fibroids from uterine leiomyosarcomas. Rare Tumors. 2025;17:20363613251327080. doi: 10.1177/20363613251327080 40291343 PMC12033571

[21] Kusunoki S, Terao Y, Ujihira T, Fujino K, Kaneda H, Kimura M, et al. Efficacy of PET/CT to exclude leiomyoma in patients with lesions suspicious for uterine sarcoma on MRI. Taiwan J Obstet Gynecol. 2017;56(4):508–13. doi: 10.1016/j.tjog.2017.05.003 28805609

[22] Hensley ML, Chavan SS, Solit DB, Murali R, Soslow R, Chiang S, et al. Genomic Landscape of Uterine Sarcomas Defined Through Prospective Clinical Sequencing. Clin Cancer Res. 2020;26(14):3881–8. doi: 10.1158/1078-0432.CCR-19-3959 32299819 PMC7367750

[23] Kim J, Williams A, Noh H, Jasper EA, Jones SH, Jaworski JA, et al. Genome-wide meta-analysis identifies novel risk loci for uterine fibroids within and across multiple ancestry groups. Nat Commun. 2025;16(1):2273. doi: 10.1038/s41467-025-57483-5 40050615 PMC11885530

[24] Przybyl J, Spans L, Lum DA, Zhu S, Vennam S, Forgó E, et al. Detection of Circulating Tumor DNA in Patients With Uterine Leiomyomas. JCO Precis Oncol. 2019;3:PO.18.00409. doi: 10.1200/po.18.00409 32232185 PMC7105159

[25] Wan JCM, Mughal TI, Razavi P, Dawson S-J, Moss EL, Govindan R, et al. Liquid biopsies for residual disease and recurrence. Med. 2021;2(12):1292–313. doi: 10.1016/j.medj.2021.11.001 35590147

[26] Moss EL, Gorsia DN, Collins A, Sandhu P, Foreman N, Gore A, et al. Utility of Circulating Tumor DNA for Detection and Monitoring of Endometrial Cancer Recurrence and Progression. Cancers (Basel). 2020;12(8):2231. doi: 10.3390/cancers12082231 32785174 PMC7463944

[27] Soberanis Pina P, Clemens K, Bubie A, Grant B, Haynes G, Zhang N, et al. Genomic Landscape of ctDNA and Real-World Outcomes in Advanced Endometrial Cancer. Clin Cancer Res. 2024;30(24):5657–65. doi: 10.1158/1078-0432.CCR-24-2105 39417689 PMC11647206

[28] Jamieson A, McConechy MK, Lum A, Senz J, Dowhy T, Huntsman DG, et al. Selective utilization of circulating tumor DNA testing enables disease monitoring in endometrial and ovarian carcinomas. J Gynecol Oncol. 2025;36(1):e5. doi: 10.3802/jgo.2025.36.e5 38909641 PMC11791001

[29] Recio F, Scalise CB, Loar P, Lumish M, Berman T, Peddada A, et al. Post-surgical ctDNA-based molecular residual disease detection in patients with stage I uterine malignancies. Gynecol Oncol. 2024;182:63–9. doi: 10.1016/j.ygyno.2023.12.025 38262240

[30] Chitrakar A, Ashmore A, Bujkiewicz S, Wheaton L, Pepper C, Guttery DS, et al. Blood-based biomarkers to diagnose and monitor recurrent endometrial cancer: A systematic review and meta-analysis. 2025. doi: 10.1101/2025.08.28.25333862

[31] Zhou M, Bui N, Rathore R, Sudhaman S, George GV, Malashevich AK, et al. Feasibility of Longitudinal ctDNA Assessment in Patients with Uterine and Extra-Uterine Leiomyosarcoma. Cancers (Basel). 2022;15(1):157. doi: 10.3390/cancers15010157 36612153 PMC9818540

[32] Arshad J, Barreto-Coelho P, Jonczak E, Espejo A, D’Amato G, Trent JC. Identification of Genetic Alterations by Circulating Tumor DNA in Leiomyosarcoma: A Molecular Analysis of 73 Patients. J Immunother Precis Oncol. 2020;3(2):64–8. doi: 10.36401/JIPO-20-3 36751526 PMC9179395

[33] Gressel GM, Maggi EC, Harmon BE, Ye KQ, Kuo DYS, Dolan SM, et al. Low molecular weight serum cell-free DNA concentration is associated with clinicopathologic indices of poor prognosis in women with uterine cancer. J Transl Med. 2020;18(1):323. doi: 10.1186/s12967-020-02493-8 32854748 PMC7457235

[34] Yokoi A, Matsuzaki J, Yamamoto Y, Tate K, Yoneoka Y, Shimizu H, et al. Serum microRNA profile enables preoperative diagnosis of uterine leiomyosarcoma. Cancer Sci. 2019;110(12):3718–26. doi: 10.1111/cas.14215 31599471 PMC6890430

[35] Relton A, Collins A, Guttery DS, Gorsia DN, McDermott HJ, Moss EL. Patient acceptability of circulating tumour DNA testing in endometrial cancer follow-up. Eur J Cancer Care (Engl). 2021;30(4):e13429. doi: 10.1111/ecc.13429 33616269 PMC11475366

[36] Chan A-W, Boutron I, Hopewell S, Moher D, Schulz KF, Collins GS, et al. SPIRIT 2025 statement: updated guideline for protocols of randomised trials. BMJ. 2025;389:e081477. doi: 10.1136/bmj-2024-081477 40294953 PMC12035670

[37] Braun V, Clarke V. Thematic analysis. London: Sage. 2022.

[38] Doll KM, Pike M, Alson J, Williams P, Carey E, Stürmer T, et al. Endometrial Thickness as Diagnostic Triage for Endometrial Cancer Among Black Individuals. JAMA Oncol. 2024;10(8):1068–76. doi: 10.1001/jamaoncol.2024.1891 38935372 PMC11211989

[39] Moss EL, Teece L, Darko N. Uterine cancer mortality and Black women: time to act. Lancet Oncol. 2023;24(6):586–8. doi: 10.1016/S1470-2045(23)00113-4 37269839

[40] Darko N, Millet N, Usman A, Teece L, Moss EL. Exploring the perspectives of underrepresented voices: Perceptions and experiences of uterine cancer for black African, Caribbean, black British, and mixed-black women in the UK to develop strategies for early symptom presentation. Gynecol Oncol. 2024;180:132–8. doi: 10.1016/j.ygyno.2023.11.030 38091772

[41] Chitrakar A, Darko N, Millet N, Weekes S, Dhanota H, Lawrence A. You need to know: evaluation of a uterine cancer awareness campaign for Black and Asian ethnic minority women in North East London. BMJ Public Health 2025;3:e002475. doi: 10.1136/bmjph-2024-002475 PMC12458616 41001240

[42] Chitrakar A, Darko N, Moss EL. Seeing red.? Co-design of a culturally tailored uterine cancer awareness intervention for Asian and Black ethnic minority groups. Gynecol Oncol Rep. 2025;58:101690. doi: 10.1016/j.gore.2025.101690 40034378 PMC11872401

[43] Hayes AJ, Nixon IF, Strauss DC, Seddon BM, Desai A, Benson C, et al. UK guidelines for the management of soft tissue sarcomas. Br J Cancer. 2025;132(1):11–31. doi: 10.1038/s41416-024-02674-y 38734790 PMC11724041

[44] Van Houdt WJ, Schrijver AM, Cohen-Hallaleh RB, Memos N, Fotiadis N, Smith MJ, et al. Needle tract seeding following core biopsies in retroperitoneal sarcoma. Eur J Surg Oncol. 2017;43(9):1740–5. doi: 10.1016/j.ejso.2017.06.009 28754227

[45] Gounder MM, Agaram NP, Trabucco SE, Robinson V, Ferraro RA, Millis SZ, et al. Clinical genomic profiling in the management of patients with soft tissue and bone sarcoma. Nat Commun. 2022;13(1):3406. doi: 10.1038/s41467-022-30496-0 35705558 PMC9200814

[46] Smith J, Zawaideh JP, Sahin H, Freeman S, Bolton H, Addley HC. Differentiating uterine sarcoma from leiomyoma: BET1T2ER Check!. Br J Radiol. 2021;94(1125):20201332. doi: 10.1259/bjr.20201332 33684303 PMC9327746

[47] Malek M, Rahmani M, Seyyed Ebrahimi SM, Tabibian E, Alidoosti A, Rahimifar P, et al. Investigating the diagnostic value of quantitative parameters based on T2-weighted and contrast-enhanced MRI with psoas muscle and outer myometrium as internal references for differentiating uterine sarcomas from leiomyomas at 3T MRI. Cancer Imaging. 2019;19(1):20. doi: 10.1186/s40644-019-0206-8 30935419 PMC6444554

[48] Nakagawa M, Nakaura T, Namimoto T, Iyama Y, Kidoh M, Hirata K, et al. A multiparametric MRI-based machine learning to distinguish between uterine sarcoma and benign leiomyoma: comparison with 18F-FDG PET/CT. Clin Radiol. 2019;74(2):167.e1-167.e7. doi: 10.1016/j.crad.2018.10.010 30471748

[49] Malek M, Gity M, Alidoosti A, Oghabian Z, Rahimifar P, Seyed Ebrahimi SM, et al. A machine learning approach for distinguishing uterine sarcoma from leiomyomas based on perfusion weighted MRI parameters. Eur J Radiol. 2019;110:203–11. doi: 10.1016/j.ejrad.2018.11.009 30599861

